# Adaptive changes in the DNA damage response during skeletal muscle cell differentiation

**DOI:** 10.3389/fcell.2023.1239138

**Published:** 2023-11-27

**Authors:** Inês Faleiro, Ana I. Afonso, André Balsinha, Beatriz Lucas, Robert M. Martin, Edgar R. Gomes, Sérgio F. de Almeida

**Affiliations:** Instituto de Medicina Molecular João Lobo Antunes, Faculdade de Medicina da Universidade de Lisboa, Lisboa, Portugal

**Keywords:** DNA damage response, cellular differentiation, skeletal muscle, cell cycle, cell death

## Abstract

DNA double-strand breaks (DSBs) trigger specialized cellular mechanisms that collectively form the DNA damage response (DDR). In proliferating cells, the DDR serves the function of mending DNA breaks and satisfying the cell-cycle checkpoints. Distinct goals exist in differentiated cells that are postmitotic and do not face cell-cycle checkpoints. Nonetheless, the distinctive requirements and mechanistic details of the DDR in differentiated cells are still poorly understood. In this study, we set an *in vitro* differentiation model of human skeletal muscle myoblasts into multinucleated myotubes that allowed monitoring DDR dynamics during cell differentiation. Our results demonstrate that myotubes have a prolonged DDR, which is nonetheless competent to repair DSBs and render them significantly more resistant to cell death than their progenitors. Using live-cell microscopy and single-molecule kinetic measurements of transcriptional activity, we observed that myotubes respond to DNA damage by rapidly and transiently suppressing global gene expression and rewiring the epigenetic landscape of the damaged nucleus. Our findings provide novel insights into the DDR dynamics during cellular differentiation and shed light on the strategy employed by human skeletal muscle to preserve the integrity of the genetic information and sustain long-term organ function after DNA damage.

## 1 Introduction

Maintaining the integrity of the genome is a fundamental requirement for the proper development and functioning of organisms. Cells have evolved sophisticated DNA repair mechanisms to counteract the challenges posed by genotoxic agents, which can induce DNA damage and compromise cellular homeostasis (Vítor et al., 2020). In addition to DNA repair mechanisms, DNA damage in proliferating cells causes the activation of cell cycle checkpoints (Vítor et al., 2020). The progression of the cell cycle is controlled by cyclins and cyclin-dependent kinases (CDKs). Cyclins serve as regulatory proteins that bind to and activate CDKs, leading to the phosphorylation of target proteins involved in driving cell cycle progression. When DNA damage is detected at any of these checkpoints, the cell cycle can be paused, allowing time for the repair processes to take place before proceeding. The main checkpoints are the G1/S checkpoint, the intra-S phase checkpoint, and the G2/M checkpoint (Ciccia and Elledge, 2010)**.** These checkpoints act as temporary brakes, halting cell division to allow time for DNA repair before resuming proliferation. Collectively, the DNA repair and cell cycle checkpoint mechanisms activated in response to a DNA lesion are termed the DNA damage response (DDR). DNA double-strand breaks (DSBs) stand out as the most detrimental of all DNA lesions. In order to fix DSBs, cells typically rely on either homologous recombination (HR) or non-homologous end joining (NHEJ) as their primary repair pathways (Vítor et al., 2020). HR is a high-fidelity DNA repair pathway that minimizes the risk of mutations by using homologous sequences as templates for repair. As the preferred homologous sequence for HR is found in the sister chromatid, this DNA repair mechanism is typically restricted to the S and G2 phases of the cell cycle of proliferating cells. NHEJ is a more rapid, yet less reliable, mechanism that repairs DSBs throughout the entire cell cycle of all cells (Ciccia and Elledge, 2010). Two key proteins involved in this decision-making process are 53BP1 and BRCA1 (Swift et al., 2021). 53BP1 is predominantly enriched in DSB foci during the G0/G1 phase of the cell cycle, preventing resection of the break to inhibit HR. As the cells transition to the S phase, BRCA1 is recruited to the DNA break (Chapman et al., 2012). BRCA1 promotes DNA end resection and commits cells to DNA repair by HR.

Besides the cell cycle, other factors influence the decision between HR and NHEJ, including the genomic location of the DSB and the local chromatin structure. Additionally, the gene transcriptional activity influences the repair pathway to be used (Price and D’Andrea, 2013). For instance, DSBs in genomic regions decorated with histone H3 trimethylated at lysine 36 (H3K36me3), a post-translational modification found in actively transcribed genes, are preferentially repaired by HR (Aymard et al., 2014; Carvalho et al., 2014; Pfister et al., 2014).

Whereas the response of proliferating cells to DNA damage has been thoroughly investigated, our knowledge of how terminally differentiated cells, such as skeletal muscle myotubes, handle genotoxic insults is still limited. When cells enter a postmitotic state, they no longer engage cell cycle checkpoints upon encountering DNA damage and lack sister chromatids, the preferred DNA templates for HR. Nevertheless, they remain transcriptionally active and require mechanisms to safeguard the genome integrity throughout their lifespan. While it is well-established that proliferating cells can undergo programmed cell death as a protective measure against DNA damage-induced genomic instability (Ciccia and Elledge, 2010), it remains unclear whether the DDR similarly causes the death of terminally differentiated cells. This question gains particular significance due to the observed resistance of differentiated tissues to apoptotic stimuli, a characteristic potentially linked to the limited self-regenerative potential of these cells (Walsh et al., 1997; Latella et al., 2004).

Here, we aimed to investigate the DDR in proliferating and terminally differentiated cells. We focused on the skeletal muscle lineage, where myoblasts undergo cell division, while terminally differentiated myotubes remain in a post-mitotic non-dividing state (Chal and Pourquié, 2017). During myogenesis, mononucleated myoblasts fuse into multinucleated myotubes and subsequently assemble into fully differentiated skeletal muscle myofibers (Chal and Pourquié, 2017). This process is tightly regulated by the expression of myogenic transcription factors, which define the gene expression program that drives differentiation (Esteves de Lima and Relaix, 2021).

Our investigation of the DDR in myoblasts before and after differentiation into myotubes revealed distinct kinetics of DNA repair and different susceptibility to DNA damage-induced cell death amongst the two cell types. We observed that myotubes have an extended DDR, which is competent to repair DSBs and render them significantly more resistant to cell death than myoblasts. Our findings provide valuable insights into the molecular mechanisms that govern the balance between genome integrity and tissue homeostasis in multicellular organisms.

## 2 Results

### 2.1 *In vitro* differentiation of human myoblasts into multinucleated myotubes

**FIGURE 1:**
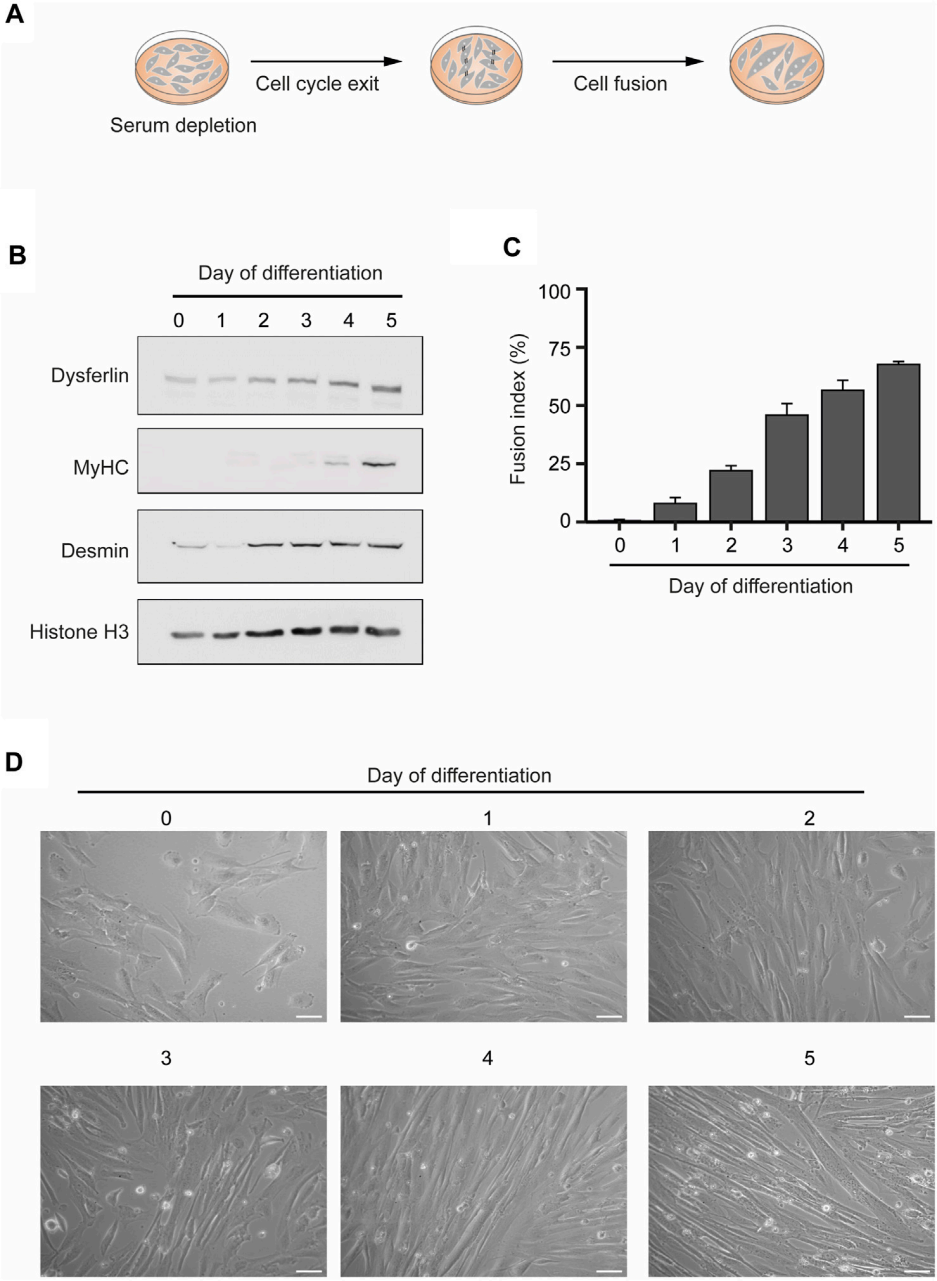
*In vitro* differentiation of human myoblasts into multinucleated skeletal muscle myotubes. **(A)**. Schematic representation of the differentiation process. Myoblast differentiation is induced by serum starvation when the cell culture is 80%–90% confluent. **(B)**. The levels of the specific skeletal muscle differentiation markers Dysferlin, Myosin heavy chain (MyHC) and Desmin were analyzed by western blot in total protein extracts of myoblasts undergoing differentiation. Histone H3 served as a loading control. Data are representative of 3 individual experiments. **(C)**. Fusion index throughout the differentiation process. Data are representative of 3 individual experiments. Error bars show the mean and standard deviation. **(D)**. Representative microscopy phase contrast images of myoblasts during myoblasts differentiation into multinucleated myotubes. Scale bar 50 µm.

To obtain human myotubes cellular cultures, we differentiated human KM155 myoblasts through serum depletion of 80%–90% confluent cultures (Mamchaoui et al., 2011) (Figure 1A). These conditions trigger a network of transcription factors that control skeletal muscle development and drive the expression of muscle-specific genes (Braun and Gautel, 2011). The increased expression of a subset of these genes - Dysferlin, Myosin Heavy Chain (MyHC), and Desmin—confirmed the successful *in vitro* differentiation of the KM155 myoblasts into myotubes (Figure 1B). While Dysferlin and Desmin are early differentiation markers and their protein levels increased after 2–3 days, MyHC is expressed in mature myotubes and, in agreement, it was detected after 4–5 days only (Figure 1B). To further confirm the efficiency of the myogenic differentiation, we estimated the percentage of nuclei belonging to cells possessing three or more nuclei over the total number of nuclei counted, a parameter termed fusion index. As multinucleation is a trademark of myotubes, the observed increasing fusion index is indicative of a successful commitment of myoblasts differentiation into myotubes (Figure 1C). Phase contrast microscopy imaging of KM155 myoblasts also reveals an increase of elongated multinucleated cells during differentiation (Figure 1D). Altogether, these data disclose the successful *in vitro* differentiation of human myoblasts into multinucleated myotubes.

### 2.2 Differentiated skeletal muscle cells have a longer DNA damage response

**FIGURE 2:**
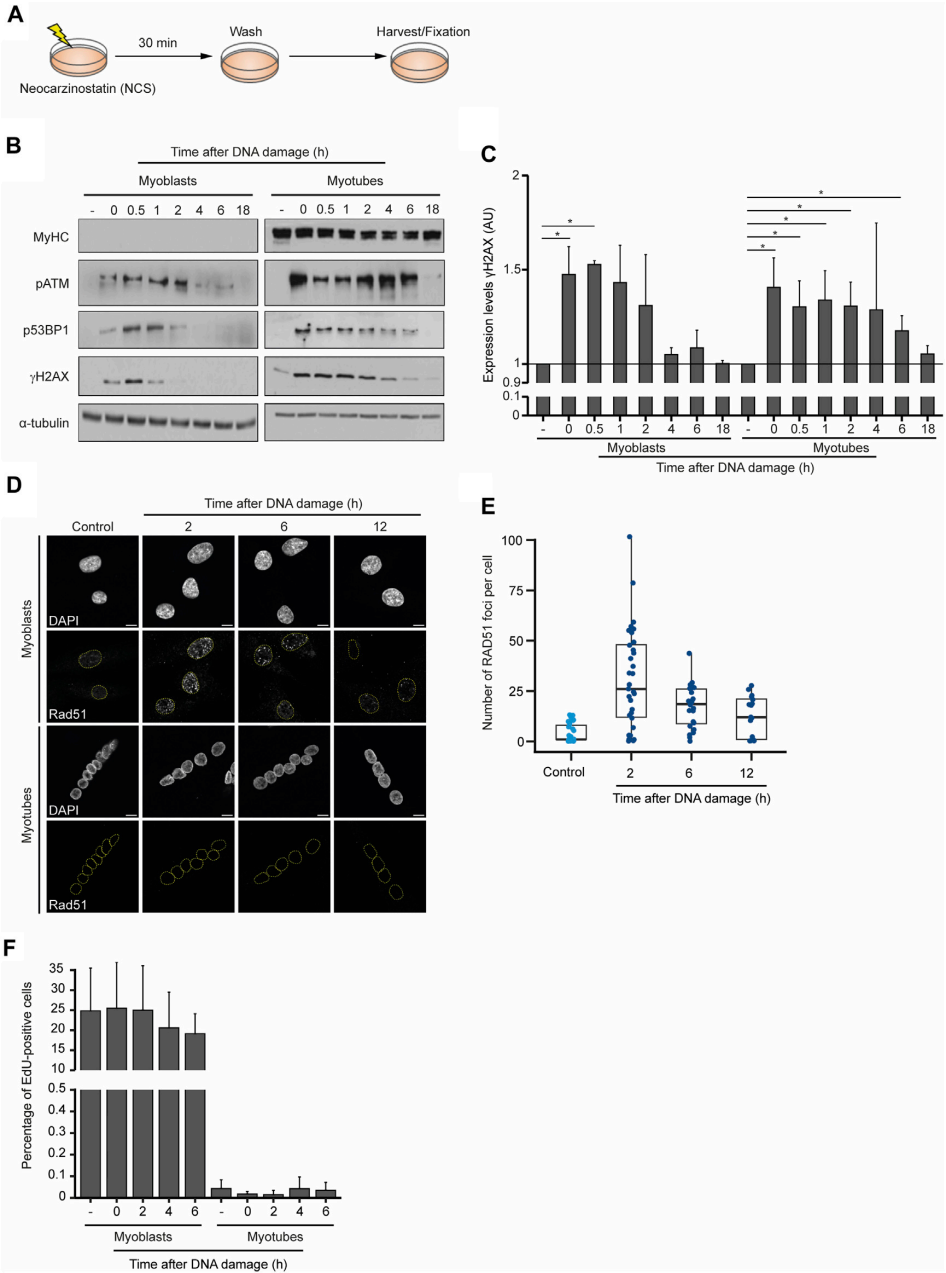
Dynamics of the DDR in skeletal muscle cells. **(A)** DNA damage was induced in myoblasts (proliferative medium) and myotubes (differentiation medium) with neocarzinostatin (NCS) for 30 min and cells were allowed to recover for the indicated time points before staining. **(B)** Western blot analysis of DDR factors in myoblasts and myotubes after NCS-induced DNA damage. α-tubulin and MyHC serve as loading and cell differentiation controls, respectively. Levels of phosphorylated ATM (pATM), 53BP1 (p53BP1) and H2AX (gH2AX) are shown. Data are representative of 5 individual experiments. **(C)** Quantification of the relative expression of gH2AX levels from the western blot data. **(D)** Skeletal muscle myotubes do not repair DSBs by homologous recombination. RAD51 immunofluorescence in myoblasts and myotubes. DAPI stains DNA, revealing the single nucleus of myoblasts and multiple nuclei in each myotube. Scale bar 10 μm. **(E)** Quantification of RAD51 foci of myoblasts upon DNA damage induction. Foci were counted in a minimum of 15 nuclei for each experimental condition. **(F)** Quantification of the percentage of EdU positive myoblasts and myotubes treated with NCS in three independent experiments. Error bars show the mean and standard deviation.

To compare the dynamics of the DDR in myoblasts and myotubes, we monitored the levels of DNA repair factors for 18 h after the chemical induction of DSBs. Neocarzinostatin (NCS), a genotoxic agent that causes DSBs genome-wide independently of the cell cycle (Povirk, 1996), was added to myoblasts and myotubes for 30 min, after which cells were cultured for the indicated time points before fixation and western blot analyses (Figures 2A, B). As anticipated, MyHC was only detected in myotubes, revealing that DNA damage did not induce any noticeable cell reprogramming or differentiation during the experiment (Figure 2B). The phosphorylation level of ataxia telangiectasia mutated (ATM-P), P53-binding protein 1 (53BP1-P) and histone H2AX (γH2AX) served as proxies for the DDR activation (Vítor et al., 2020). Robust ATM-P was observed up to 2 h after DNA damage in myoblasts, whereas in myotubes its levels remained high for 4 h longer. In myoblasts, 53BP1-P and γH2AX were detected until 2 h after DNA damage, whereas in myotubes phosphorylation of these proteins was observed until 18 h after NCS treatment (Figure 2B). Quantification of the relative levels of γH2AX reveals that myoblasts can effectively repair the DNA within 2 h, whereas myotubes exhibit persistent DNA damage for 6 h after NCS treatment (Figure 2C). These data show that while both cell types trigger the DDR with similar kinetics, the time it takes for the clearance of DNA damage differs significantly. While myoblasts complete the DDR within 2–4 h, differentiated myotubes take at least 18 h to restore the phosphorylation levels of the inspected repair factors.

The kinetics of the two major DSB repair pathways differ significantly in agreement with the complexity of the steps necessary for their completion. Not surprisingly, the ligation of the DNA broken ends by NHEJ is significantly faster than the homology-directed repair involved in HR (Mao et al., 2008; Vítor et al., 2020). Owing to the need for a sister chromatid to serve as a template for DNA repair, HR is mostly restricted to the S and G2 phases of the cell cycle and, thus, limited to proliferating cells. Nevertheless, we wondered whether myotubes would be exceptionally dependent on HR and could reenter the cell cycle to complete DSB repair. A similar mechanism was previously observed in postmitotic neurons, where cell cycle re-activation is a critical element of the DNA damage response (Kruman et al., 2004). Analysis of RAD51 distribution in myoblasts showed the formation of numerous nuclear foci after NCS treatment, which are evocative of the assembly of nucleoprotein microfilaments required for DNA strand invasion during HR (Figures 2D, E). In contrast, myotubes lacked any detectable RAD51 foci formation 16 h after DNA damage (Figure 2D), providing strong evidence against the hypothesis that HR operates in these postmitotic skeletal muscle cells. To directly assess the cell cycle oscillations of myoblasts and myotubes in response to DNA damage we measured the incorporation of 5-Ethynyl-2′-deoxyuridine (EdU), a thymidine analogue commonly used to monitor DNA synthesis during the S phase (Salic and Mitchison, 2008). These experiments revealed that in contrast to myoblasts, which were able to proceed into the S phase as indicated by abundant EdU incorporation, myotubes did not re-enter the cell cycle during the DDR and maintained the non-proliferative condition of postmitotic cells (Figure 2F). Altogether, these results suggest that myotubes engage a lengthier DDR that does not induce cell cycle re-entry and do not rely on homology-directed DNA repair pathways.

**FIGURE 3:**
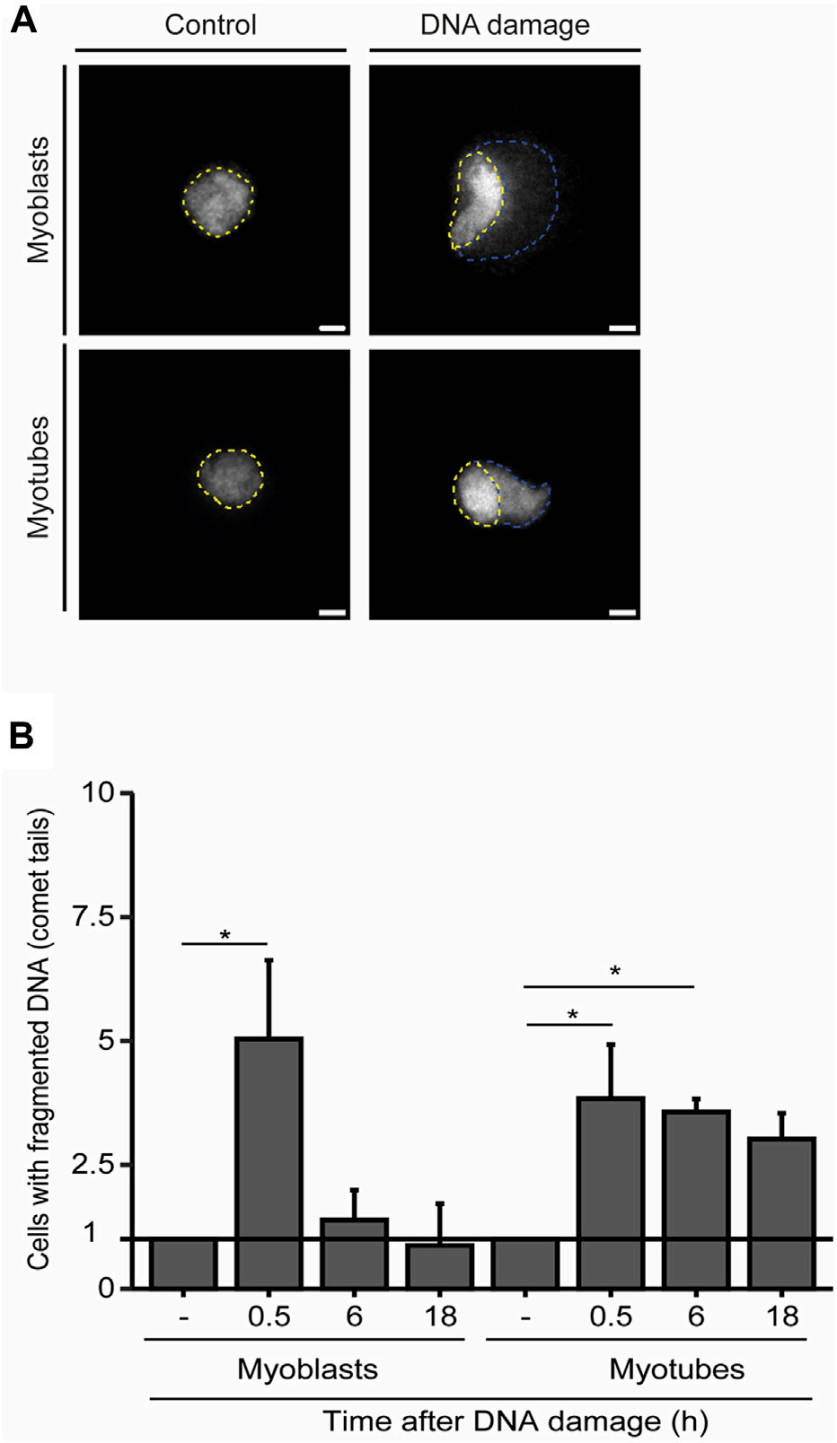
Myotubes have an extended DDR. **(A)**. Representative images of the comet assay in myoblasts (proliferative medium) and myotubes (differentiation medium). The nucleus is delineated by yellow dashed lines, while the fragmented DNA present in the comet’s tail is outlined by blue dashed lines. **(B)**. Quantification of the fraction of cells that present DNA tails after DNA damage induction with NCS. Data are normalized against the amount of non-treated cells (−) that show a comet tail. Data are representative of at least 3 individual experiments. *p < 0.05, unpaired Student’s t-test. Error bars correspond to the mean +- standard deviation.

To further explore the dynamics of DNA repair in myoblasts and myotubes we performed single-cell gel electrophoresis (comet assay), a sensitive technique for detecting DNA damage in individual cells. Our findings demonstrate that the percentage of myoblasts displaying fragmented DNA (visible as a comet’s tail), significantly diminishes within 6 h after NCS treatment, revealing that the DNA was efficiently repaired (Figures 3A, B). In contrast, we observed a greater proportion of myotubes with fragmented DNA 6 h after damage induction (Figures 3A, B). These results underscore the extended persistence of DNA lesions in myotubes compared to myoblasts.

### 2.3 Myotubes are resistant to DNA damage-induced cell death

**FIGURE 4:**
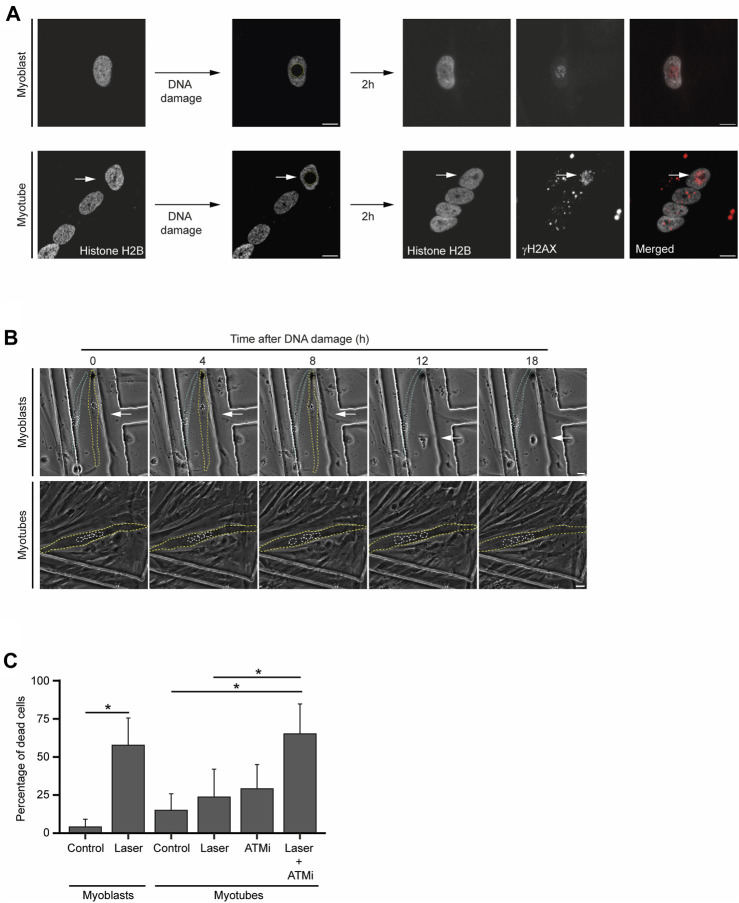
Myotubes are resistant to DNA damage-induced cell death. **(A)**. Representative images from live-cell immunofluorescence imaging with an antibody against γH2AX upon laser microirradiation of myoblasts (proliferative medium) and myotubes (differentiation medium). White arrows represent the damaged nucleus of the myotube. Scale bar 10 µm. **(B)**. Representative phase-contrast images from time-lapse live imaging experiments of myoblasts (proliferative medium) and myotubes (differentiation medium) at distinct time points upon DNA damage induction. Yellow and blue dashed lines represent cell contours. White dashed lines represent nuclei contours. White arrows represent the damaged nucleus. Scale bar 10 µm. **(C)**. Cell viability analysis of myoblasts and myotubes 24 h after DNA damage with or without treatment with an ATM inhibitor (ATMi). Data are representative of at least 3 individual experiments. *p < 0.05, unpaired Student’s t-test. Error bars show the mean and standard deviation.

In the presence of persistent DNA damage, a specific branch of the DDR eventually drives cell apoptosis (Ciccia and Elledge, 2010). To investigate how frequently the lengthier DDR observed in myotubes culminate in cell death, we set a time-lapse live-cell imaging assay upon laser microirradiation of a single nucleus of a multinucleated myotube (Figure 4A). Using this assay, we measured changes in cellular morphology characteristic of cell death, such as cell shrinkage, blebbing and fragmentation (Häcker, 2000; Saraste and Pulkki, 2000) (Figure 4B). While myoblasts displayed an extensive amount of these morphological figures in response to DNA damage, myotubes were particularly resilient and committed to cell death less frequently, as revealed by the reduced number of morphological changes (Figure 4B). Direct quantification of cell death 24 h after laser microirradiation of a single nucleus, revealed that a significant percentage of myoblasts (58%, 37 out of a total of 64 cells analysed) died upon DNA damage. In contrast, only 24% (22 out of 93) of myotubes died upon irradiation of a single nucleus. This percentage is not significantly higher than the percentage of non-irradiated cells that died during 24 h (15%, 19 out of 124) (Figure 4C). Notably, when combined with an ATM kinase inhibitor (ATMi), which hinders DDR signalling, laser irradiation led to a significant increase in myotube lethality (65%, 35 out of 54) (Figure 4C). The treatment of myotubes with the ATMi alone caused only mild cell death (29%, 56 out of 194). These findings underscore the enhanced resistance of myotubes to DNA damage-induced cell death in comparison to myoblasts and suggest that the DDR plays a role in this distinctive phenotype.

### 2.4 DNA damage-dependent epigenetic and transcriptional rewiring in myotubes

**FIGURE 5:**
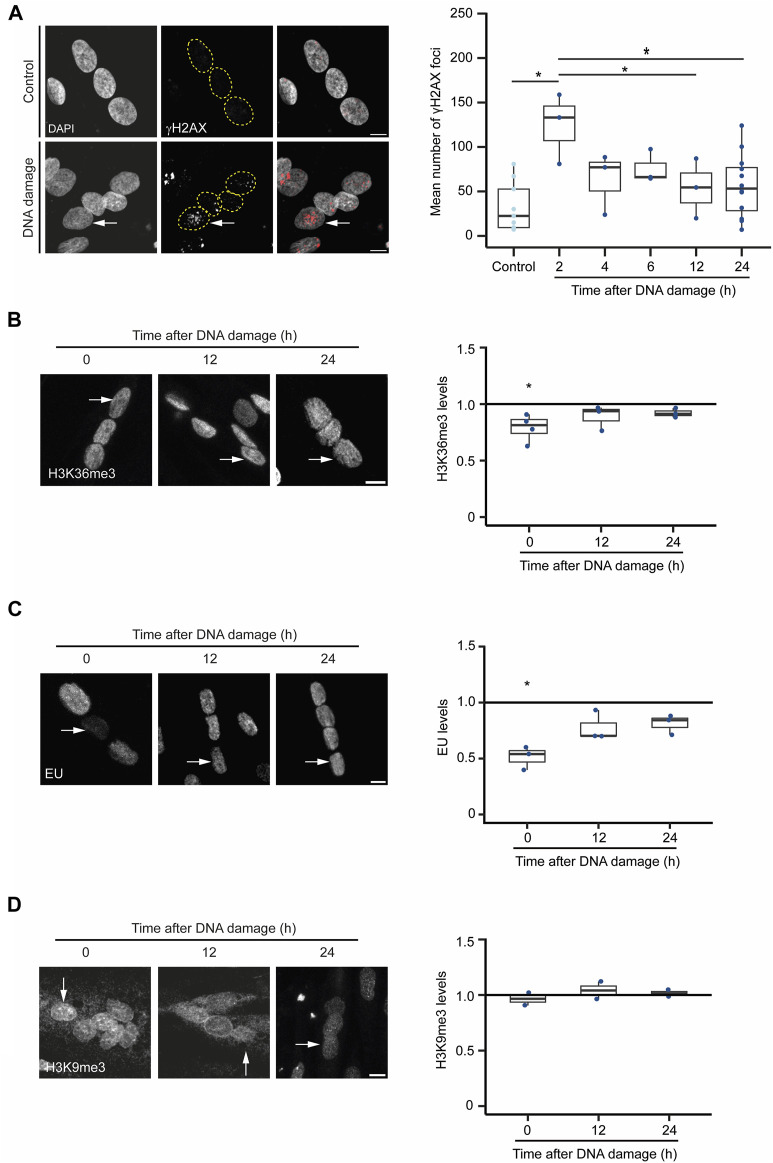
DNA damaged-induced chromatin alterations in myotubes. **(A)**. γH2AX immunofluorescence in myotubes before (control) and 12 h after DNA damage by laser microirradiation of a single nucleus (arrow). Scale bar = 10 µm. The number of γH2AX foci at different time points after DNA damage are plotted. Data are from 3 individual experiments. *p < 0.05, unpaired Student’s t-test. **(B)**. H3K36me3 immunofluorescence in myotubes before and after DNA damage by laser microirradiation of a single nucleus (arrows). Scale bar = 10 µm. The ratio of H3K36me3 levels between damaged and undamaged nuclei was calculated at 0, 12 and 24 h after DNA damage. Data are from 3 individual experiments. *p < 0.05, unpaired Student’s t-test. **(C)**. Evaluation of the levels of global nascent transcription through EU incorporation in myotubes before and 12 h after DNA damage by laser microirradiation of a single nucleus (arrows). The ratio of EU levels between damaged and undamaged nuclei was calculated at 0, 12 and 24 h after DNA damage. Scale bar = 10 µm. Data are from 3 individual experiments. *p < 0.05, unpaired Student’s t-test. **(D)**. H3K9me3 immunofluorescence in myotubes before and after DNA damage by laser microirradiation of a single nucleus (arrows). Scale bar = 10 µm. The ratio of H3K9me3 levels between damaged and undamaged nuclei was calculated at 0, 12 and 24 h after DNA damage. Data are from 2 individual experiments.

Next, we sought to investigate the hypothesis that severe DNA damage in a single nucleus of a myotube causes the inactivation of the compromised nucleus as a strategy to prevent aberrant gene expression caused by mutagenic DNA repair while preserving cell integrity. For that, we inspected whether DNA damage drives a global rewiring of the epigenetic landscape and gene expression programs upon DNA damage in myotubes. We monitored the levels of H3K36me3, a histone modification that decorates active chromatin, and 5-ethynyl uridine (EU) incorporation, a proxy for global nascent transcription, following laser microirradiation of individual nuclei in myotubes (Jao and Salic, 2008; de Almeida et al., 2011; Carvalho et al., 2014). The number of γH2AX foci, indicative of the assembly of DSB repair centres, peaked in the damaged nuclei 2 h after irradiation, returning to near-basal levels 12 h later (Figure 5A). The levels of both H3K36me3 and EU incorporation in the irradiated nuclei decreased rapidly and significantly after DNA damage, recovering to their initial levels 12 h later (Figures 5B, C). These results suggest that transcription is not permanently repressed in the damaged nuclei arguing against the view that myotubes drive whole-nuclei inactivation in response to severe DNA damage. In agreement, we did not observe any significant enrichment of H3K9me3, a histone modification found in heterochromatin, in the nuclei of cells that had been irradiated (Figure 5D).

**FIGURE 6:**
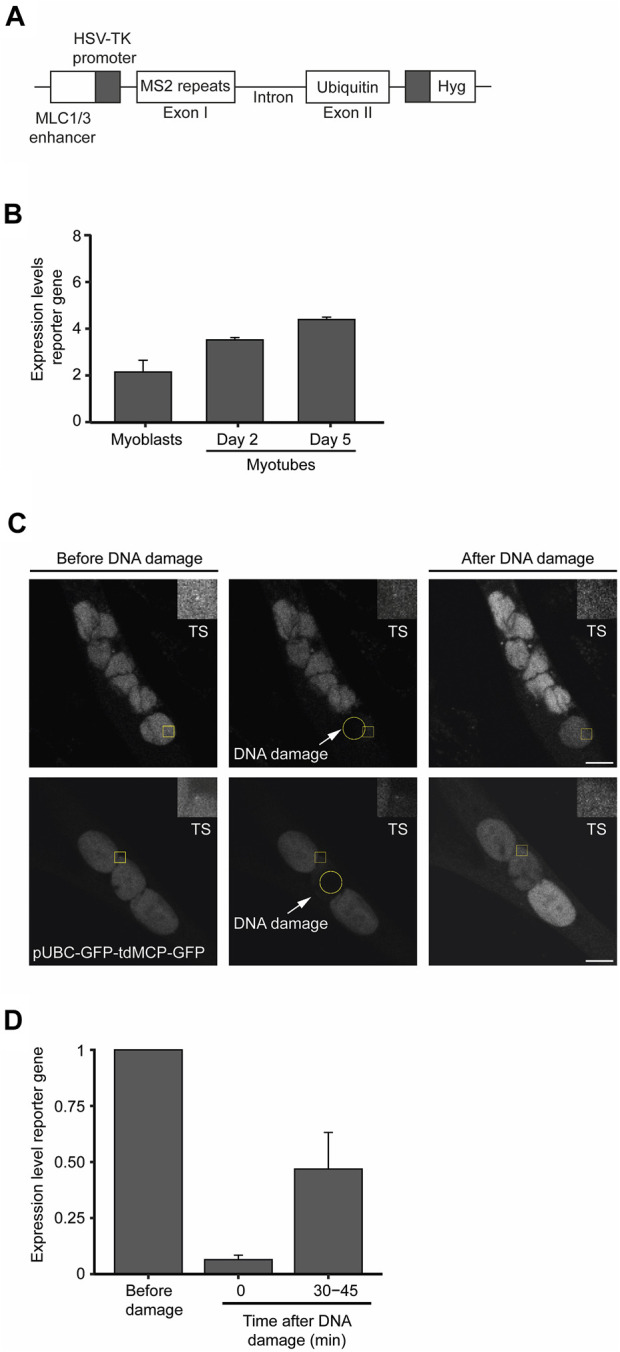
Transcription is transiently suppressed in myotubes in response to DNA damage. **(A)**. Schematic representation of the reporter gene stably integrated in the genome of KM155 myoblasts. **(B)**. Transcription levels of the reporter gene during the differentiation of myoblasts into myotubes were assessed by quantitative RT-PCR. Represented are the mean and SD from three independent experiments. AU: arbitrary units. Error bars show the mean and standard deviation. **(C)**. Representative images of myotubes expressing the reporter gene. Yellow circles represent the region where DNA damage was induced and yellow squares represent a region of interest (ROI) defined around the transcription site (TS) and zoomed in at the top right corner of the image. **(D)**. Dynamics of transcription of the reporter gene upon laser microirradiation estimated by the oscillations in the total fluorescence intensity levels measured at the TS. Error bars correspond to the mean +− standard deviation.

To measure changes in transcription dynamics after DNA damage with superior resolution and accuracy, we established a novel myoblast cell line containing a reporter gene whose expression is under the control of an upstream human myosin light chain enhancer element that is expressed following skeletal muscle differentiation (Rosenthal et al., 1990). The reporter gene was derived from the human Ubiquitin B gene and was engineered to contain 24 tandem repeats of the MS2-coding sequence within exon 1 (Figure 6A). The binding of transiently expressed MS2-GFP fusion proteins to the MS2 stem-loops in the nascent reporter gene’s transcript enables the live-cell visualization of ongoing transcription with single RNA molecule resolution (Vítor et al., 2019). Reverse transcriptase quantitative PCR (RT-qPCR) performed at different time points of the differentiation process confirmed the myotube differentiation-dependent expression of the reporter gene (Figure 6B). We then measured the kinetics of transcription of the reporter gene by live-cell microscopy following laser microirradiation of a single nucleus of myotubes. To prevent photobleaching of the MS2-GFP fluorescence, we directed the laser to a nuclear region distant from the reporter gene transcription site. Analysis of the MS2-GFP fluorescence intensity showed that transcription of the reporter gene was rapidly suppressed within a few seconds upon DNA damage, and resumed to approximately 50% of the initial levels after 30 min (Figures 6C, D). Altogether, these findings reveal rapid fluctuations of transcription rates in response to DNA damage in myotubes, suggesting that permanent or long-term interruption of gene expression is not a major DDR strategy employed by myotubes to safeguard cell integrity.

## 3 Discussion

In this study, we compared the dynamics of the DDR in human myoblasts and myotubes after the induction of DSBs. Skeletal muscle cells serve various functions, with force production and movement being particularly relevant. Importantly, the process of muscle contraction leads to endogenous DNA lesions. When muscles contract, there is a substantial energy demand and deformation of the nucleus, which cause DNA damage owing to oxidative stress or compromised integrity of the nuclear envelope, respectively (Barbieri and Sestili, 2012; Jabre et al., 2021)**.** Increased levels of DNA damage have been observed in muscle dystrophies, underscoring the importance of elucidating the DNA repair mechanisms operating in skeletal muscle cells (Bou Saada et al., 2017). Due to their multinucleated nature, we hypothesized that skeletal muscle myotubes possess the ability to specifically inactivate individual nuclei upon DNA damage. We conceive this mechanism as an effective approach to maintaining the integrity of the genetic information while ensuring the survival of the cell. Our data show that the kinetics of the DDR activation were similar in both cell types but the time it took for the clearance of DNA damage differed significantly. Myoblasts took 2–4 h to complete the DDR, whereas damaged DNA was detected in myotubes for at least 18 h. The faster kinetics observed in myoblasts is compatible with the time required to resolve DNA lesions through NHEJ (Mao et al., 2008), which are nevertheless competent to engage HR as revealed by the presence of RAD51 foci. The lack of RAD51 foci formation and EdU incorporation revealed that HR does not operate in myotubes. Moreover, we observed that these cells do not re-enter the cell cycle during the DDR. This distinguishes postmitotic cells of the skeletal muscle lineage from neurons, which can revert their cell cycle halt after DNA damage as a means to engage programmed cell death pathways (Kruman et al., 2004).

Previous studies have reported differences in the DNA repair capacity between the different cells of the mouse skeletal muscle lineage. Adult mouse skeletal stem cells repair radiation-induced DSBs more efficiently than their committed progeny (Vahidi et al., 2014). Moreover, mouse skeletal muscle myotubes are defective in base excision DNA repair, whereas mouse myoblasts can engage this pathway in response to oxidative DNA damage (Narciso et al., 2007). Taken together, these results strongly suggest that the DNA repair capacity is tightly linked to the differentiation status of the cells. The emerging consensus is that DNA repair efficiency decreases as a function of cell differentiation. A notable trait of differentiated muscle cells is their remarkable resistance to cell death (Latella et al., 2004; Vahidi et al., 2014)**.** Given that differentiated tissues constitute the majority of an organism’s body mass, and skeletal muscle cells are among the longest-living cells in the human body, impaired apoptotic signalling may represent an important mechanism to sustain organ function. The balance between the pro-apoptotic and anti-apoptotic members of the Bcl-2 family of proteins is crucial for determining the susceptibility of myotubes to apoptosis. Myotubes express high levels of anti-apoptotic proteins Bcl-2 and Bcl-xL, whereas pro-apoptotic proteins like Bcl-2-associated X protein (BAX) and Bcl-2 antagonist/killer (BAK) are typically expressed at low levels (Latella et al., 2004; Vahidi et al., 2014). Moreover, apart from their involvement in cell cycle regulation, p21 and Rb have been linked to the regulation of the apoptotic resistance exhibited by skeletal muscle cells. This suggests that the exit from the cell cycle is connected to the resistance to cell death in myotubes (Walsh and Perlman, 1997). However, the underlying mechanisms remain unclear. Notably, we observed that myotubes are remarkably resistant to DNA damage-induced cell death through an ATM-dependent mechanism. A similar resistant phenotype was also found in myotubes treated with ionizing radiation and is linked to impaired p53 phosphorylation (Latella et al., 2004).

As myotubes avoid DNA damage-induced cell death despite the prolonged duration of the DDR, alternative DSB-repair pathways should operate in these postmitotic cells. Our finding that gene expression is not permanently switched off in a single nucleus whose DNA had been damaged by irradiation, suggests that multinucleation does not endow myotubes with the capacity to selectively and permanently inactivate individual nuclei with a compromised genome. Maintaining a transcriptionally active status demands mechanisms that deal with DSBs to preserve the integrity of the genetic information. What is the mechanistic basis of such mechanisms and whether they operate exclusively in the skeletal muscle cells or are found in other differentiated tissues are important questions that emerge from this study.

Overall, this study contributes to our understanding of the molecular mechanisms that control skeletal muscle development and differentiation and provides a foundation for future research in this area. A better understanding of the DDR in myotubes will certainly expand our capacity to interpret the age-dependent deterioration of skeletal muscle function and to identify new strategies for preventing and treating skeletal muscle diseases.

## 4 Materials and methods

### 4.1 Myoblasts cell culture and differentiation induction

The KM155 cell line (Human Skeletal Myoblasts) was established by Vincent Mouly from the Institut de Myologie UPMC Université Paris France (Mamchaoui et al., 2011). The undifferentiated myoblasts were grown in a proliferative medium: Skeletal Muscle Cell Media (PromoCell C-23060) supplemented with a mix containing Fetal Calf Serum (0,05 mL/mL), Fetuin (50 μg/mL), Epidermal Growth Factor (10 ng/mL), Basic Fibroblast Growth Factor (1 ng/mL), Insulin (10 μg/mL) and Dexamethasone (0,4 μg/mL). Differentiation was induced when cells were 80%–90% confluent by serum starvation. To induce myoblast differentiation the growth medium was removed, and cells were washed twice with PBS 1x before adding the differentiation medium. The differentiation medium contained an equal quantity of Iscove’s Modified Dulbecco’s Medium (IMDM GlutaMAX™ Supplement—Alfagene 31980022) and Ham’s F-10 Nutrient Mixture (F-10) (Alfagene 41550021) supplemented with 1% ITS (I1884 Sigma Aldrich - Contains 1.0 mg/mL recombinant human insulin, 0.55 mg/mL human transferrin (substantially iron-free), and 0.5 μg/mL sodium selenite at the 100x concentration). The differentiation medium was changed every other day for 5 days. All experiments with myotubes were performed with cells maintained in the differentiation medium. Cells were maintained at 37°C in a humidified atmosphere containing 5% CO2.

### 4.2 Fusion index

The fusion index was calculated as the percentage of nuclei in myotubes (cells with three or more nuclei) relative to the total of nuclei counted. Approximately 300–500 cells were counted.

### 4.3 Transfections

Transfection of the cells was performed with Lipofectamine^®^ 3000 transfection reagent (L3000015, Invitrogen). For every transfection, two solutions were prepared. The first solution contained 125 μL of Opti-MEM Reduced Serum Medium (Opti-MEM) (31985070, Gibco) plus 1 ug of plasmid DNA and 2 μL of P3000 per 1 μg of DNA. The second solution contained the same amount of Opti-MEM and 1,5 μL of Lipofectamine 3000 reagent per each μg of DNA. The solutions were incubated for 5 min at room temperature separately, then the solutions were gently mixed, and the final mixture was incubated for 20 min at room temperature before being added to the cells. 24 h after transfection the medium was changed.

### 4.4 Plasmids and generation of reporter cells

KM155 cell line was used for genomic integration of the pMLCenh-24bcMS2-H4C-2UBB_Hyg reporter gene illustrated in Figure 6A. The reporter gene was designed based on the human ubiquitin B gene (HGNC:12463; ENST00000302182.7) comprising exon I, one intron which includes a barcode sequence and exon II with an open reading frame of two head-to-tail ubiquitin units fused in frame to an N-terminal HA tag (synthesis by GeneArt, Invitrogen). A sequence containing 24 MS2 stem-loop sequence tandem repeats with five nonidentical spacers serving as barcodes/primer binding sites described before was ligated in sense direction into exon I of the reporter gene [described in (24)]. The reporter gene construct was then ligated into the backbone of a plasmid containing a minimal herpes simplex virus thymidine kinase (HSV-TK) promoter under the control of an upstream human myosin light chain enhancer element (MLC1/3F), providing exclusive gene expression in differentiated myogenic cells such as myotubes. The resulting plasmid was named pMLCenh. To allow the selection of cells with stable reporter gene integration we inserted a hygromycin resistance gene (Hyg) under the control of an HSV-TK promoter into the reporter gene plasmid. The Hyg gene was amplified by PCR from pTK-Hyg (Clontech, Takara Bio Inc.) using primers which contain a 5′ NdeI and a 3′ BshTI site. The PCR product was digested with the respective enzymes and ligated into the same sites in a new multiple cloning site inserted into the DraIII site in pMLCenh-24bcMS2-2UBB by a short oligonucleotide ligation before. The final plasmid was then linearized by MlsI digest and the DNA directly was purified using the NZYGelpure kit (NZYtech, Lisbon, Portugal). The KM155 myoblast cell line was subsequently transfected with 1 µg of the linearized plasmid and 24 h later selection started by adding 200 μg/mL Hygromycin (Invivogen) to the growth medium. During the selection of stably transfected myoblast clones, we used 50/50% volume of fresh growth medium and sterile filtered pre-conditioned medium harvested as the supernatant of 48 h KM155 myoblast cell cultures. After 2–3 weeks, a mixed culture of all selected reporter gene clones of the KM155 MS2-H4C2UBB cell line was established and used in the experiments described.

### 4.5 Live-cell microscopy imaging

Cells were grown in gridded glass bottom dishes (81166—iBidi—µ-Dish 35 mm, high Grid-500 ibiTreat) to facilitate the localization of the cells. Zeiss LSM 880 confocal point-scanning microscope (Carl Zeiss) was used to visualize transfected cells. Phase contrast time-lapse live imaging cell experiments were performed using the Nikon Eclipse Ti microscope using a 20x/Air objective (NA = 0.45). To visualize the transcription site, KM155 MS2-H4C2UBB cells were transfected with the pUBC-GFP-tdMCP-GFP plasmid. This plasmid encodes for two GFP molecules tagged to two MS2 proteins allowing the visualization of the transcription site. We measured the total fluorescence intensity (TFI) levels of the transcription site of cells using the ImageJ software (v1.53t) as follows: the TFI of the transcription site was measured as well as four regions of surrounding areas. Doing the average of these four values, it was obtained the average TFI value of the background of the images. Then, the value of the background was subtracted from the value of the respective transcription site to reduce the noise or any artefacts in the images due to photobleaching. Due to heterogeneous levels of expression of the plasmid used to visualize the reporter gene, the TFI values of each nucleus were normalized to the values before DNA damage.

### 4.6 DNA damage induction

Myotubes or myoblasts were treated with 250 ng/mL of NCS (Sigma N9162) for 30 min, washed with PBS-1x and maintained in differentiation or growth medium, respectively. DNA damage in single nuclei of differentiated human skeletal muscle cells was induced by UV laser microirradiation under a state-of-the-art confocal laser-scanning microscope (Zeiss LSM880). We carried out experiments to assess the protocol under two conditions: one with cells sensitized using Hoechst prior to laser irradiation and the other without sensitization. In the former case, cells were treated with Hoechst (1:1000) for 10 min, washed twice with PBS-1X, and a fresh differentiation medium was added to the cells. In the presence of Hoechst sensitization, we observed a diffuse staining of γH2AX throughout the cells, as opposed to the localized foci formation observed without sensitization (Supplementary Figure S1). Consequently, we decided to proceed with laser irradiation without any sensitization.

A single nucleus was irradiated by 3 pulses with 5 min intervals using 100% emission from a 30 mW Diode 405 nm Laser (Acquisition setting: size 512 × 512, zoom 2, ROI circle dimension 60). KM155 cells were transfected with pUBC-H2B-GFP to allow nuclei visualization during the laser-irradiation experiments and cells with 3–6 nuclei were chosen to facilitate relocation of the same nuclei. The pUBC-H2B-GFP plasmid was previously generated in our lab [described in (Martin and Cardoso, 2010)]. In combinatorial treatments, 2.5 uM of the ATM inhibitor KU-55933 (ab120637) was added before damage induction. At distinct time points, cells were fixed for immunofluorescence or collected for western blot.

### 4.7 Immunofluorescence

Cells grown on coverslips or gridded glass bottom dishes were fixed with 3.7% paraformaldehyde for 10 min at room temperature. The myoblasts and myotubes were then permeabilized with 0.5% or 1% Triton X-100/PBS, respectively, for 10 min. Cells were then incubated at room temperature for 30 min in a blocking solution of PBS-BSA 2%. Incubation with primary and secondary antibodies was performed for 1h at 37°C. All washing steps were done with PBS containing 0.05% (vol/vol) Tween 20. Finally, nuclear staining was performed using Hoechst 33342 (Thermo Fisher Scientific H1399) or DAPI (09542; Sigma Aldrich) at a dilution of 1:1000 in PBS 1× for 10 min at room temperature, and cells were mounted with Fluoromount G (Lab Clinics 00-4958-02). The following primary antibodies were used: RAD51 (Abcam/ab213), γH2AX (Millipore/05-636, phosphoSer139 H2AX), H3K36me3 (Abcam/ab9050), H3K9me3 (Abcam/ab8898). The following secondary antibodies were used: Alexa Fluor 647 anti-mouse (Thermo Fisher Scientific/A-21236) and Cy3 anti-rabbit (Bethyl/A120-201C3). Images were acquired using the Zeiss LSM 710 or LSM 880 point scanning confocal microscopes using a 40x or 63x/1.4 oil immersion objective, respectively. Images were acquired with stacking acquisition and generation of maximum intensity projection images. Image analysis was performed using ImageJ (v1.53t). For γH2AX and RAD51 analysis, we counted the number of foci per nuclei. For the analysis of γH2AX levels in myotubes upon DNA damage, we considered the number of γH2AX foci of the damaged nuclei. For non-damaged myotubes, we performed the mean of γH2AX foci of all nuclei. For the analysis of H3K36me3 and H3K9me3 levels, we measured the total nucleoplasmic fluorescence intensity (TFI) per nuclei. Then, for each cell, the level of the damaged nucleus was normalized to the levels of non-damaged nuclei.

### 4.8 5-ethynyl-2′-deoxyuridine (EdU) incorporation

Myoblasts and myotubes were incubated with EdU (10 μM) during different time point intervals after treatment with NCS as described above. Cells without treatment were incubated with EdU for 2 h to measure the basal levels of EdU incorporation. Cells were washed with cold PBS 1X, scraped out and centrifuged at 4000 rpm for 5 min at 4°C. The process of fixation, permeabilization and click-iT reaction was done according to the manufacturer’s protocol (C-10425 Click-iT EdU Flow Cytometry Assay Kit protocol, Thermo Fisher Scientific). Cells were immediately analysed with BD Accuri C6. The results are presented as the percentage of positive cells. Flow cytometry data was analysed using the FlowJo software (v10.8.0). To evaluate the feasibility of myotube analysis via flow cytometry, we conducted immunofluorescence staining on differentiated myotubes, following the aforementioned protocol. To that end, we evaluated the expression of MyHC, a myotube differentiation marker. Cells were stained using an anti-MyHC primary antibody (Developmental Studies Hybridoma Bank, MF20) and the Alexa Fluor 488 anti-mouse secondary antibody (Thermo A21202). Following the staining procedure, the cells were collected and subjected to analysis using the BD Accuri C6 cytometer. Additionally, differentiated myotubes were harvested through trypsinization, washed with cold 1x-PBS, and subsequently centrifuged for 5 min at 500 g, after which the supernatant was removed. These cells were then fixed for 15 min at −20°C in 1 mL of ice-cold 70% EtOH. After a subsequent centrifugation at 600 g for 5 min, the supernatant was discarded. The cells were then resuspended and incubated with PI (5 µg) diluted in PBS-1X for 10 min before undergoing analysis using the Amnis imaging cytometer. We were able to identify the expression of MyHC in myotubes using the Accuri cytometer (Supplementary Figure S2A). Furthermore, images obtained through the Amnis imaging cytometer demonstrate the existence of multinucleated cells within the culture (Supplementary Figure S2B).

### 4.9 5-ethynyl uridine (EU) staining

Myoblasts and myotubes were grown on gridded glass bottom dishes and incubated for 1h (37°C, 5% CO2) with EU from the Click-iT RNA Alexa Fluor 594 imaging kit (C10330, Invitrogen). Cells were fixed with 3,7% formaldehyde in PBS 1× for 15 min at room temperature and permeabilized with 1% Triton X-100 in PBS 1× for 10 min at room temperature. The Click-It reaction using a fluorescent azide (Alexa Fluor 594 azide) was then performed according to the manufacturer’s instructions (30min at room temperature, protected from light). Finally, nuclear staining was performed with Hoechst 33342 at a dilution of 1:1000 in PBS 1× for 10 min at room temperature, and cells were mounted with Fluoromount G (Lab Clinics 00-4958-02). Cells were imaged using a point-scanning confocal microscope Zeiss LSM 880, 63×/1.4 oil immersion, with stacking acquisition. Nucleoplasmic fluorescence intensity measurements were performed using ImageJ. The fluorescence intensity of the damaged nucleus was normalized to a non-damaged nucleus of the same cell.

### 4.10 RNA isolation and quantitative RT-PCR

Total RNA isolation from KM155 MS2-H4C2UBB was performed using TRIzol reagent (15596018, Invitrogen) at distinct days during differentiation. To prepare cDNA from the extracted RNA we used the NZY First-Strand cDNA Synthesis Kit (NZYTech). All qPCR reactions were performed in a ViiA 7 Real-Time PCR system (Applied Biosystems) using the PowerUp SYBR Green Master Mix (A25918, Applied Biosystems). Relative RNA levels were quantified using the 2^−ΔΔCT^ formula using GAPDH expression levels as reference. The following primers were used: H4C2UBB-ExI-Fw - ACG​GCC​AGT​CTA​GGT​TAG​GT, H4C2UBB-ExII-Rev—TGGCTCCATACAGCAACCAG, GAPDH-Fw—GAAGGTGGAGGTCGGAGTC and GAPDH-Rv—GAAGATGGTGATGGGATTTC.

### 4.11 Western blot

To prepare total cell protein extracts, cells were washed twice with PBS 1x and lysed in Laemmli Buffer 2X (80 mM pH 6.8, 16% glycerol, 4.5% SDS, 450 mM DTT, 0.01% bromophenol blue and water, with 100 U benzonase (Sigma Aldrich) and 1% (v/v) MgCl2). The lysates were incubated at room temperature for 20 min and boiled for 5 min at 100°C. Then, equal amounts of protein extract and NZYColour Protein Marker (NZYTech) were loaded on the wells of an 8% or 12% polyacrylamide gel in parallel with a colour protein marker (MB09002, NZYTech). The gels were resolved in SDS-polyacrylamide gel electrophoresis (SDS-PAGE) using a Mini-PROTEAN Tetra Electrophoresis System (Biorad). Next, proteins were dry transferred to a nitrocellulose membrane with an iBlot2 System (Thermo Fisher Scientific). Then, the membrane was blocked for 1 h at room temperature, with a blocking solution composed of 5% (m/v) milk in PBS 1x containing 0,05% (v/v) Tween 20 (PBS-T). The membrane was then incubated overnight at 4°C with primary antibodies diluted in 5% blocking solution, washed 3 times for 5 min with PBS-T and incubated for 1 h at room temperature with the appropriate horseradish peroxidase (HRP)–coupled secondary antibody. Protein detection was achieved using enhanced chemiluminescence substrates (RPN 2134, Amersham and 34096, Thermo Fisher Scientific). The following antibodies were used: Dysferlin (Abcam, ab124684), MyHC (Developmental Studies Hybridoma Bank, MF20), Desmin (Dako, D3), Histone H3 total (Abcam, ab1791), ATM-P (Rockland, 200-301-400s), 53BP1-P (Cell Signaling, 2675), γH2AX (Millipore, 05-636), α-tubulin (Sigma, T5168).

### 4.12 Single-cell gel electrophoresis (comet assay)

Myoblasts and myotubes were treated with NCS and collected at distinct time points upon damage induction. In this study, we employed the commercially available comet assay kit from Abcam and followed the protocol provided within the kit (ab238544). Images were acquired using the LSM 980 point scanning confocal microscopes using a 20× objective. Images were acquired with stacking acquisition and generation of maximum intensity projection images. Image analysis was performed using ImageJ (v1.53t).

## 5 Data representation and statistics

All graphs were plotted using R and formatted in Adobe Illustrator (Adobe Inc., San Jose, CA, United States). Statistical tests were performed using R. Unpaired Student’s t-test was performed to infer statistical significance. Statistical significance is represented as follows: *p < 0.05; ns, no significance. Error bars represent the standard deviation.

## Data Availability

The raw data supporting the conclusion of this article will be made available by the authors, without undue reservation.

## Supplementary material

SUPPLEMENTARY FIGURE S1 Laser microirradiation induces generalized DNA damage in myotubes. γH2AX immunofluorescence of myotubes subjected to DNA damage by laser microirradiation with or without Hoechst sensitization. White arrows represent the damaged nucleus of the myotube.

SUPPLEMENTARY FIGURE S2 Flow cytometry analysis of myotubes. **(A)**. Representative histogram of the flow cytometry analysis of myotubes stained for the differentiation marker myosin heavy chain (MyHC). Cells were selected according to their size, as determined by the forward scattering area (FSC-A), and granularity, assessed through the side scattering area (SSC-A). The expression of MyHC was determined by assessing the mean fluorescence intensity (MFI) of the fluorescence signal. **(B)**. Representative images of myotubes acquired using the Amnis imaging cytometer. PI stains the nuclei. BF: brightfield. Scale bar = 10 µm.
