# Supplementary figures and images for: Adaptive changes in the DNA damage response during skeletal muscle cell differentiation
Source: Front Cell Dev Biol. 2023 Nov 27;11:1239138. doi: 10.3389/fcell.2023.1239138 (PMC10711097; doi:10.3389/fcell.2023.1239138)

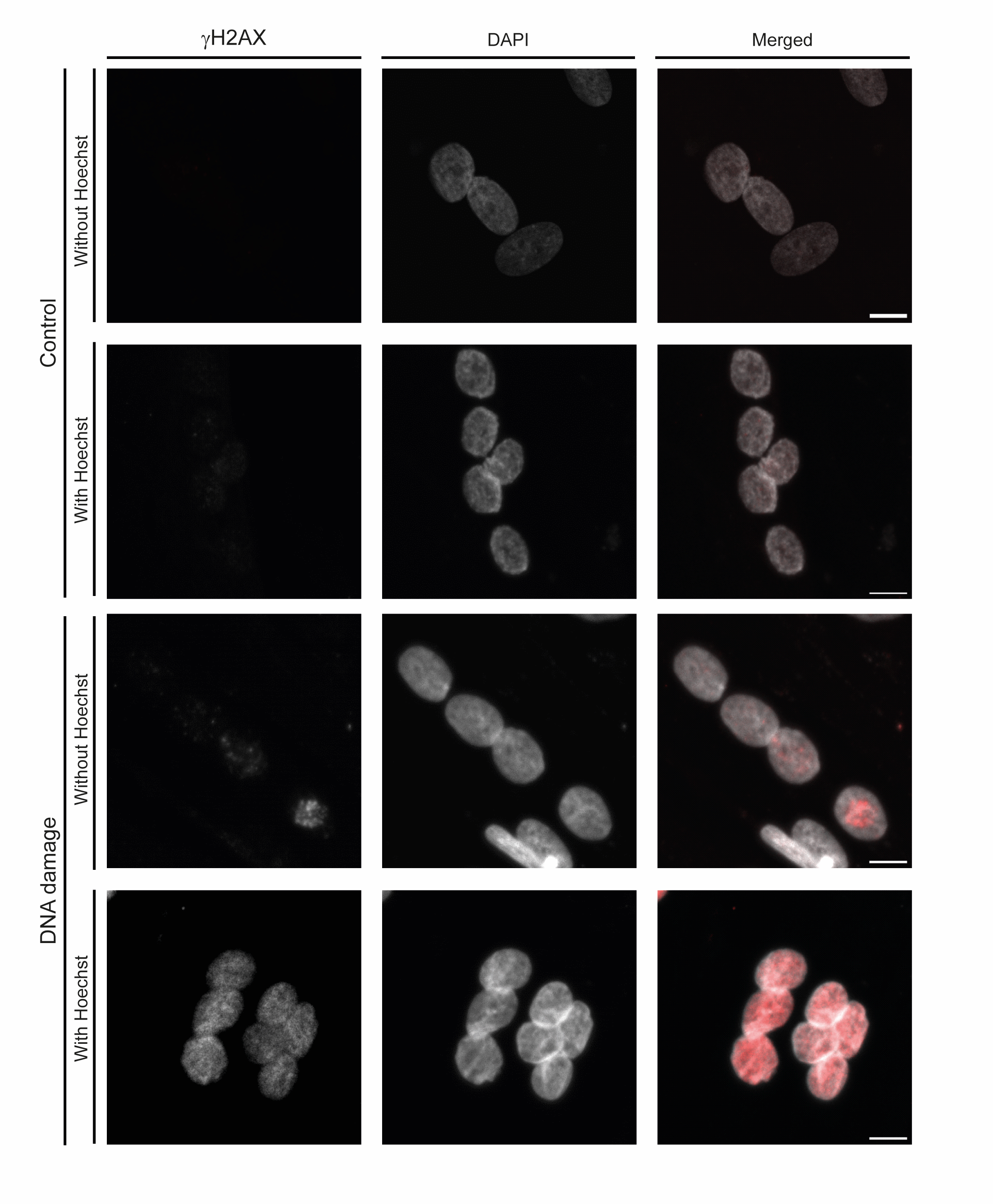

Supplement: Supplementary file 1 [file Image1.JPEG]

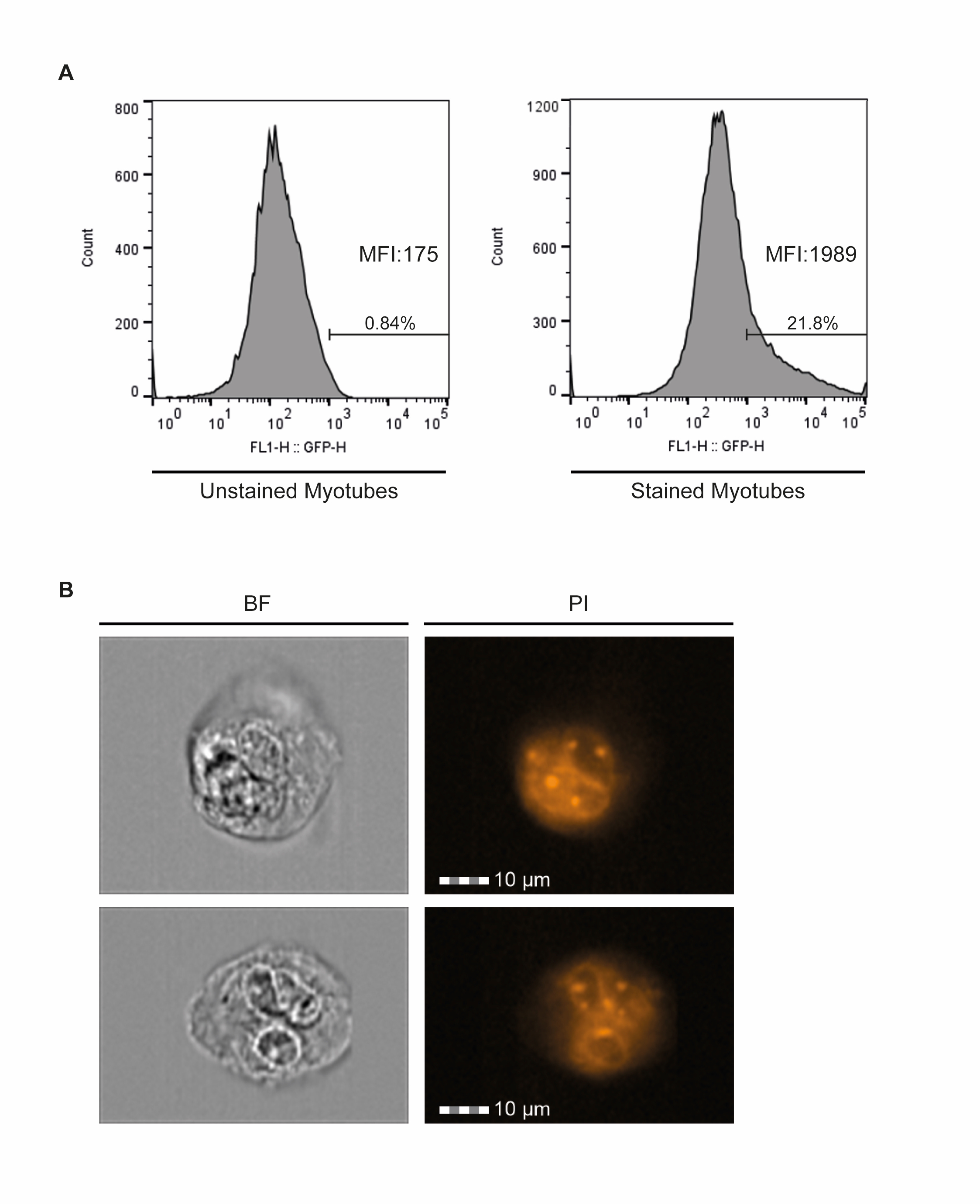

Supplement: Supplementary file 2 [file Image2.JPEG]
